# Vestibular Rehabilitation in Saudi Arabia: Practice, Knowledge, and Beliefs of Physical Therapists

**DOI:** 10.3390/jcm14072295

**Published:** 2025-03-27

**Authors:** Maryam ALShammari, Doaa S. ALSharif, Mishal M. Aldaihan, Susan L. Whitney

**Affiliations:** 1 Department of Cochlear Implants, Hafr Albatin Central Hospital, Ministry of Health, Hafar Al Batin 39513, Saudi Arabia; maryamea@moh.gov.sa; 2 Department of Physical Therapy, College of Applied Medical Sciences, Taif University, Taif 21974, Saudi Arabia; 3 Department of Rehabilitation Science, College of Applied Medical Sciences, King Saud University, Riyadh 11451, Saudi Arabia; mishaldaihan@ksu.edu.sa; 4 King Salman Center for Disability Research, Riyadh 11614, Saudi Arabia; 5 Department of Physical Therapy, School of Health and Rehabilitation Sciences, University of Pittsburgh, Pittsburgh, PA 15260, USA; whitney@pitt.edu

**Keywords:** vestibular rehabilitation, dizziness, knowledge, physical therapy, Saudi Arabia

## Abstract

**Background**: Vestibular physical therapy is a specific type of exercise intervention that is designed to treat symptoms caused by vestibular disorders and to enhance postural control. The level of expertise in the practice of vestibular rehabilitation therapy (VRT) varies widely around the world. The practice of VRT is advanced in some countries, yet practice may be less advanced in others. No previous studies have evaluated the knowledge and beliefs of vestibular rehabilitation in the Kingdom of Saudi Arabia (KSA) to date. **Objective**. This study aimed to evaluate the knowledge and beliefs about VRT among physical therapists in the KSA. **Methods**: We used an electronic cross-sectional survey containing 25 questions and divided into four sections: demographics, clinical experience, vestibular rehabilitation knowledge, and physical therapists’ beliefs. The survey was answered by 219 licensed physical therapists. **Results**: Of the 219 responders, 59 (27%) physical therapists reported having experience with VRT. A total of 119 (54%) participants felt confident talking to other medical members when managing persons with dizziness. Only 59 (26%) clinicians were using vestibular assessment techniques, and 103 (47%) clinicians used VRT if they had patients with vestibular disorders. The majority of physical therapists believed in the efficacy of vestibular rehabilitation. **Conclusions**: Although the majority of physical therapists consider vestibular rehabilitation a crucial aspect of their practice, they acknowledge having limited knowledge of vestibular disorders and treatment techniques.

## 1. Introduction

Dizziness affects a person’s quality of life, and it is often accompanied by an increased healthcare burden [1,2]. The prevalence of dizziness in older adults 65 years or older is around 30% [3], and in younger adults, dizziness has been reported in 23% of the population [4]. The prevalence of dizziness in Saudi Arabia is estimated to be around 43% [5]. The most common cause of dizziness is due to peripheral vestibular disorders [6,7]. In the absence of medical or surgical interventions for patients with vestibular disorders, vestibular rehabilitation therapy (VRT) is the preferred treatment choice either pre- or post-operatively [8].

Vestibular rehabilitation therapy (VRT) is an intervention that is contingent on exercise to enhance postural control and to decrease symptoms caused by vestibular disorders [9,10,11]. VRT has positive effects on dizziness, vertigo, gait, risk of falls, activities of daily living, and quality of life [12,13,14,15,16,17]. VRT is effective in persons living with benign paroxysmal positional vertigo [18,19], unilateral vestibular hypofunction [12,20,21], vestibular migraine [22], and persistent postural perceptual dizziness [23].

The level of practice of VRT varies widely around the globe. While the practice of VRT in some countries is advanced, e.g, Australia and Sweden, where therapists provide VRT, it may be more challenging to obtain VRT in less developed countries [24]. In Japan, few physical therapists utilize VRT for patients with dizziness because it is not reimbursed [25]. Additionally, in Korea, the otolaryngologists, neurologists, and other medical staff are the health providers who perform vestibular rehabilitation education for people living with dizziness [26,27]. Practice varies widely across the globe, although the Barany Society has recently developed a curriculum that includes what they perceive is entry level and advanced practice skills for physical therapists [28]. The variability of VRT might be attributed to limitations in knowledge and skills [24,28].

However, it is unknown how many physical therapy practitioners practice vestibular rehabilitation in the Kingdom of Saudi Arabia. Alyahya and Kashoo reported that physicians’ lack of awareness about the role of physical therapy in managing patients with vestibular disorders is common in Saudi Arabia [29]. A recent study by Albalwi et al. found that Saudi Arabian physical therapists lack knowledge, referrals, equipment, and training to conduct vestibular examinations, which leads to dissatisfaction with the current vestibular rehabilitation system [30]. Indeed, we hypothesize that in Saudi Arabia, few physical therapists are practicing and specializing in vestibular rehabilitation. Thus, this study aims to describe the knowledge and beliefs of physical therapy practitioners about vestibular rehabilitation in the Kingdom of Saudi Arabia.

## 2. Materials and Methods

This cross-sectional survey was conducted online in Saudi Arabia in the period between March and August 2021. The survey was approved by the Local Ethical Committee for Bioethics of the King Abdulaziz City for Science and Technology (no. H-05-FT-083) prior to distribution. Convenience sampling was used to recruit participants. The inclusion criteria required that the physical therapists be currently practicing full-time or part-time, be licensed by the Saudi Commission for Health Specialties (SCFHS), and that they fully completed the survey. Exclusion criteria included physical therapists who were full-time academicians, physical therapist students, interns, or physical therapists who were not currently practicing. All participants read the electronic Informed Consent Form and voluntarily agreed to participate before proceeding to the next section of the online survey, which included the study questionnaire. No personal information such as name or governmental identification number was required to participate.

### 2.1. Survey Development

The authors developed an electronic survey based on concepts from similar previous studies [31,32] to describe knowledge and beliefs of vestibular rehabilitation among physical therapist professionals. The survey was developed in cooperation with two academic professors with expertise in vestibular rehabilitation and research methodology. Once the survey was finalized, ten physical therapists were asked to pilot test the survey as local expert stakeholders to provide feedback on the questionnaire and improve its clarity. After a consensus among experts and stakeholders was achieved, the questionnaire and its purpose were distributed.

Close-ended questions were utilized to make the survey more time-efficient and easier to analyze. The survey (see Appendix A) consisted of 25 questions, and it was divided into four sections: (1) demographics; (2) clinical experience; (3) vestibular rehabilitation knowledge; and (4) physical therapists` beliefs.

### 2.2. Survey Distribution

The survey was distributed as an electronic Qualtrics survey (Qualtrics LLC, Provo, Utah) that was disseminated to 500 physical therapists who were currently practicing in a clinical setting in Saudi Arabia, and 391 responses were recorded. Of the 391 respondents, 219 completed the survey. During the five-month survey period (March–August 2021), the survey was distributed eight times on a weekly basis. Each participant was allowed to respond once using their device IP identification number. Participants’ answers were collected and stored in a password-protected electronic format. All responses were anonymized. No one other than the authors had access to the collected data.

### 2.3. Data Analysis

Data were downloaded from Qualtrics into Excel and analyzed using R version 4.0.4 (R Foundation for Statistical Computing, Vienna, Austria). We used descriptive statistics (categorical variables were presented in frequencies and percentages) to analyze demographic characteristics of the participants (e.g., gender, nationality, highest earned degree), clinical characteristics, beliefs, sources of acquired vestibular rehabilitation knowledge, vestibular rehabilitation assessment and treatment techniques, and respondents’ knowledge and attitudes about vestibular rehabilitation. Moreover, we used the chi-square test to identify the association between some variables (e.g., highest earned degree, primary working setting, experience in the physical therapy (PT) field, if physicians referred patients with vestibular disorders to physical therapists, and experience in VRT). A *p*-value of 0.05 was considered statistically significant. The required sample size was 471, which was estimated based on a previous study with a similar objective [31].

## 3. Results

Demographic data. As shown in Table 1, of the respondents from the survey, 59% were male, while 41% were female. A total of 95% of the 219 respondents were Saudis. The majority of participants (62%) reported a bachelor’s degree as their highest earned degree, while the lowest (4%) reported a doctorate (PhD, DSc, EdD). A majority of participants (45%) were employees of the Ministry of Health, followed by physical therapists in private hospitals (25%), and private practice employees represented the minority.

Clinical characteristics. Of the 219 complete responders, 27% (*n* = 59) of the physical therapists reported having experience with VRT; however, 59% out of the 59 physical therapists who reported VRT experience had less than one year of experience (Table 2). A total of 83% of the 59 participants (49 participants) reported having didactic and some practical training in vestibular rehabilitation.

Beliefs about vestibular rehabilitation. A total of 86% out of 219 participants believed that physical therapists should be a member of a multidisciplinary team that manages patients with vestibular disorders, while only 30% were not sure of the physical therapy role in managing vestibular disorders. Only 26% of respondents believed that physical therapists are not able to treat patients with vestibular disorders, while the majority (87%) believed that physical therapists are able to treat vestibular disorders. According to the survey, 89% of respondents believed vestibular disorders significantly impacted health, and 88% believed that physical therapists trained in vestibular rehabilitation can evaluate patients with vestibular disorders (see Table 3).

Knowledge and attitudes about vestibular rehabilitation. Of those 219 responders with vestibular rehabilitation experience, 54% learned about VRT during workshops and conferences, and 31% learned from their Saudi school’s physical therapy educational program, while only 19% gained their skills from clinical experience (Table 4). Regarding physicians’ referrals, 76% of physical therapists reported that physicians did not refer patients with vestibular disorders. When managing individuals with dizziness, 54% of participants felt confident in talking to other medical professionals (Table 2).

About 27% of practitioners were using vestibular assessment in their clinical practice. They reported that the supine roll testing, 68%, and the Dix–Hallpike testing, 61%, were the most utilized assessment techniques, and the least-used bedside test was perilymphatic fistula, 10% (Table 5). A total of 47% out of 219 responders reported having used vestibular rehabilitation techniques, such as canalith repositioning maneuvers (42%) and optokinetic exercise (18%), for interventions. The most common exercise provided was a VORx1 exercise (50%). The VORx1 exercise is where a person moves their head in either pitch or yaw while focusing on a target (Table 6).

**Figure 1:**
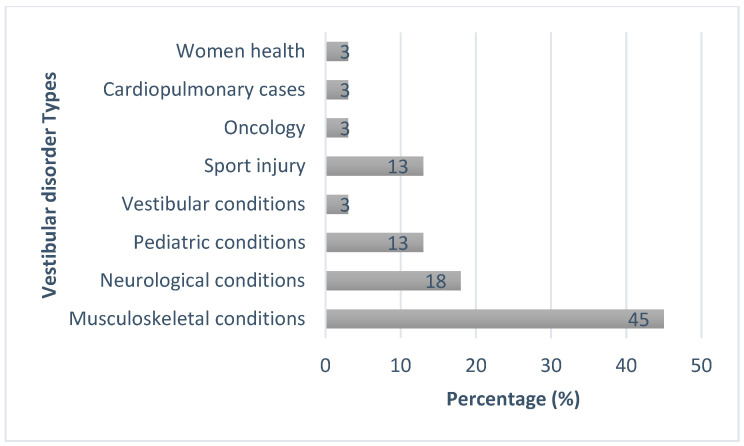
Monthly time spent treating various diagnoses by participants (*n* = 219) *. * Responses are presented as *n* (%).

Regarding knowledge of vestibular pathologies among all responders in the survey, multiple sclerosis (88%), traumatic brain injury (88%), and stroke (78%) were reported as the most known diagnoses seen by physical therapists who might report dizziness. Fifty-five percent of the physical therapists reported that they did not have knowledge about the treatment of persons with vestibular migraine, and 75% agreed that they would not feel comfortable treating persons with vestibular migraine. Moreover, 58% reported that they were aware of the diagnosis of BPPV, but only 24% of the respondents felt comfortable treating persons with BPPV, and 27% were comfortable treating persons with cervical dizziness (Table 7). Furthermore, our results showed that the minority of practitioners spent their time treating patients with oncologic, cardiopulmonary, and vestibular conditions, while the majority of them were managing patients with musculoskeletal conditions (Figure 1).

In analyzing the association between the highest earned degree, primary working setting, experience in the PT field, if physicians referred patients with vestibular disorders, and experience in VRT, the chi-square test revealed a statistically significant relationship only between if physicians referred patients with vestibular disorders to PT and experience in VRT, where χ^2^ (1, N = 219) = 39.72, *p*-value < 0.001. Of those who had received referrals from physicians, 54% had experience in vestibular rehabilitation, and 13% had no experience in vestibular rehabilitation (Table 8).

## 4. Discussion

In our current study, we were able to record the level of knowledge, beliefs, and attitudes of physical therapists about vestibular rehabilitation practice in Saudi Arabia. The low percentage of therapists who are practicing VRT in Saudi Arabia is reflective of their lack of knowledge about the examination and treatment of persons living with dizziness. Practitioners who were treating individuals with vestibular disorders often had less than a year of experience in the field. Similar to a previous study by Albalwi et al., our results showed that the majority of vestibular physical therapy practitioners gained their knowledge about VRT from workshops and conferences [30]. This indicates that seminars and workshops are the primary sources of learning about the VRT profession [30], followed by Saudi schools’ physical therapy educational program. Previous studies showed that the level of knowledge that therapists have in VRT is related to the level of knowledge and education they received during their undergraduate or graduate level training [28,31]. Our results indicate that the physical therapists who received post-graduate education reported knowledge of and confirmed practicing VRT. In Saudi Arabia, no accredited VRT curriculum is required as part of entry-level training in physical therapist educational programs.

Regarding the VRT level of knowledge, the study found that 44% reported a lack of knowledge as a barrier to not practicing VRT among physiotherapists. Another study showed that approximately 45% of physiotherapists are aware of VRT, but their practical experience and formal training are still limited, with only 30% reporting any hands-on experience with VRT techniques [29]. Research from multiple countries indicates that the level of knowledge and experience with VRT is often limited. A study in the United States found that only 50% of physiotherapists reported having formal training in VRT, with about 40% of them stating they had never used VRT in clinical practice [33]. A similar study in the United Kingdom revealed that 60% of physiotherapists were aware of VRT, but only 35% had received any formal training in the technique [34]. In Australia, a survey showed that 55% of physiotherapists knew about VRT, but only 25% felt confident in delivering it as part of their treatment approach [35]. These findings show that while physiotherapists across various regions are generally aware of VRT, a significant portion still lack formal training and practical experience in its application. This aligns with the findings of our study, which suggests that awareness and knowledge of VRT in Saudi Arabia may not be fully satisfactory, compared to international standards. Therefore, it highlights the need for targeted educational programs and improved training for physiotherapists, both in KSA and globally, to bridge this knowledge gap and ensure effective treatment for patients with vestibular disorders.

We found that the majority of physical therapists do not receive vestibular rehabilitation referrals from physicians. A recent study conducted in Saudi Arabia reported that vestibular rehabilitation services in physiotherapy clinics are underutilized due to limited referrals from physicians. The dearth of referrals was reportedly because physicians assumed that physical therapists do not have the expertise to treat persons with vestibular disorders [29]. Additionally, our results showed a high percentage of physical therapists who reported that they do not treat individuals with VM, BPPV, and cervical dizziness. Since these disorders can be managed by VRT, those individuals will either look to other clinicians or end up giving up and withdrawing from care. There are potential cost savings when persons are treated early with vestibular disorders, as there is a significant reduction in fall rates post-physical therapy intervention in persons with dizziness [36].

Falls and long-term dizziness affect work and leisure activities; additionally, there are costs associated with additional medical assessment and examination [37]. Therefore, we recommend that a vestibular rehabilitation curriculum be developed that could be extracted from the baseline knowledge and skills documents that were defined in the Barany Societies entry-level curricula. The curriculum should minimally introduce basic level knowledge of the vestibular system, vestibular disorders, and neuropathology, in addition to knowledge of the therapists’ own capacities and limitations. The decision to treat or refer individuals with vestibular disorders is a critical skill for best management. Stewart et al. developed models of care whereby physical therapists assist in the diagnosis of vestibular disorders in the emergency department in Queensland [38]. As physical therapists acquire additional knowledge in Saudi Arabia, care should improve for persons living with vestibular disorders, and the economic burden should be reduced on the healthcare system.

There are several limitations to this study. The sample is slightly underpowered based on the power analysis, so the results should be considered with some caution. Only 27% of the respondents had any experience treating persons with vestibular disorders, so the responses about what type of interventions were used should be viewed carefully. However, the 44% response rate overall for the survey is considered average for an online survey [39]. The persons who selected to respond to the survey may have biased the survey, as they may have had greater interest in the survey than the non-responders.

## 5. Conclusions

Most physical therapists identified vestibular rehabilitation as an essential part of their practice yet reported that they had a limited understanding of vestibular disorders and VRT. The development of high-quality educational programs about vestibular rehabilitation is essential to improving care for persons living with dizziness in Saudi Arabia. Providing quality education about vestibular rehabilitation in physical therapist entry level educational programs and providing opportunities for post-entry level education is essential to begin to change practice in Saudi Arabia.

## Figures and Tables

**Table 1:** Demographics of the physical therapist respondents from Saudi Arabia (*n* = 219).

	Characteristics	Measurement *
Gender	Male	128 (59%)
	Female	91 (41%)
Nationality	Saudi	208 (95%)
	Others	11 (5%)
Highest earned degree	PT Bachelor’s	135 (62%)
	Doctor of Physical Therapy (DPT)	18 (8%)
	Master’s	57 (26%)
	Terminal Doctorate (PhD, DSc, EdD)	9 (4%)
Primary work setting	Military/National Guard/Security hospital	32 (15%)
	Ministry of Health hospital	100 (45%)
	Private hospital/clinic	55 (25%)
	Private practice	3 (1%)
	University hospital	19 (9%)
	Other	10 (5%)

* Measurements are presented as *n* (%).

**Table 2:** Physical therapist clinical characteristics (*n* = 219) *.

Items	Responders with VRT Experience	Responders with no VRT Experience
Number of participants	59 (27%)	160 (73%)
Years of experience in PT field:		
Less than 1 year	4 (7%)	21 (13%)
1–5 years	30 (51%)	53 (33%)
6–10 years	14 (24%)	37 (23%)
Over 10 years	11 (18%)	49 (31%)
Years of experience in VRT:		
Less than 1 year	35 (59%)	
1–5 years	19 (32%)	
6–10 years	2 (3%)	
Over 10 years	3 (5%)	
Have physicians ever referred patients with vestibular disorders to you?		
Yes	32 (54%)	21 (13%)
No	27 (46%)	139 (87%)
Do you see one patient with vestibular disorder or more per month?		
No	36 (61%)	138 (86%)
Yes	23 (39%)	22 (14%)
If yes, what is the average number of patients with vestibular disorders you see per month?		
1	11 (48%)	13 (58%)
2–5	10 (44%)	7 (32%)
6–10	1 (4%)	1 (5%)
Over 10	1 (4%)	1 (5%)
Are you confident in communicating with other medical team members (e.g., ear, nose, and throat physicians) in managing patients with dizziness?		
Yes	41 (70%)	78 (49%)
No	2 (3%)	36 (22%)
Not sure	16 (27%)	46 (29%)
Reasons for not treating patients with vestibular disorders (only responders with no experience in vestibular rehabilitation) †		
Lack of knowledge		70 (44%)
Lack of confidence		16 (10%)
Lack of experience		69 (43%)
Physicians do not refer patients to me		74 (46%)
Persons with dizziness are managed by another specialty e.g., audiologist, ear, nose, and throat physicians		63 (39%)
I am not allowed to treat persons with dizziness in my practice		8 (5%)

* Responses are presented as *n* (%). † Multiple selections were allowed.

**Table 3:** Saudi Arabian physical therapist beliefs about vestibular rehabilitation (*n* = 219) *.

Item	Yes	No	Not sure
Do you believe that physical therapists should be a member of a multidisciplinary team that manages patients with vestibular disorders? Do you believe that physical therapists are able to treat patients with vestibular disorders? Do you believe that vestibular disorders have a significant impact on the health of individuals and society?	189 (86%)	−	30 (14%)
	191 (87%)	2 (1%)	26 (12%)
	195 (89%)	2 (1%)	22 (10%)
	192 (88%)	1 (0%)	26 (12%)

* Responses are presented as *n* (%).

**Table 4:** Sources of acquired vestibular rehabilitation knowledge (all respondents with and without vestibular rehabilitation experience who only had didactic and some practical training in vestibular rehabilitation) (*n* = 113)) *.

Item: If Yes, From Where You Have Obtained Your Knowledge About Vestibular Rehabilitation? †	No. (%)
In my physical therapy curriculum as part of my entry-level education in Saudi Arabia	35 (31%)
In my physical therapy curriculum as part of my entry-level education outside Saudi Arabia.	10 (9%)
During my clinical experience as a staff member	21 (19%)
During my internship as a student in Saudi Arabia	15 (13%)
During my internship as a student outside Saudi Arabia	3 (3%)
Through workshops and conferences	61 (54%)
Received the experience from an expert colleague	15 (13%)

* Responses are presented as *n* (%). † Multiple selections were allowed.

**Table 5:** Vestibular assessment techniques that the physical therapist is confident in implementing in his/her clinical practice (only respondents who answered this question) (*n* = 59) *,†.

Item ‡	No. (%)
Dix–Hallpike test	36 (61%)
Supine roll test	40 (68%)
Side-lying test	28 (47%)
Head Impulse Test	26 (44%)
Dynamic Visual Acuity Test	24 (41%)
Oculomotor examination (pursuits, saccades, gaze holding)	25 (42%)
Head Shaking Nystagmus test	35 (59%)
Perilymphatic fistula test	6 (10%)
VOR Cancellation	20 (34%)
Modified Clinical Test of Sensory Interaction and Balance (M-CTSIB)	12 (20%)

* Responses are presented as *n* (%). † Only respondents who answered this question were reported. ‡ Multiple selections were allowed.

**Table 6:** Vestibular rehabilitation treatment techniques that the physical therapist is confident in implementing in his/her clinical practice (*n* = 103) *,†.

Item ‡	No. (%)
Canalith Repositioning Maneuver	43 (42%)
Semont Maneuver	27 (26%)
Gufoni Maneuver	22 (21%)
Barbecue Roll Maneuver	28 (27%)
Imaginary target exercise	31 (30%)
Vestibulo-ocular reflex 1 (VOR 1)	51 (50%)
Vestibulo-ocular reflex 2 (VOR 2)	45 (44%)
Gaze shifts between two targets	48 (47%)
Optokinetic stimulation	19 (18%)

* Responses are presented as *n* (%). † Only respondents who answered this question were reported. ‡ Multiple selections were allowed.

**Table 7:** Saudi Arabian physical therapist respondents’ knowledge and attitudes about vestibular rehabilitation (*n* = 219) *.

Item	Knowledge		Attitudes	
	Know †	Do Not Know	Treat	Do Not Treat
Benign paroxysmal positional vertigo (BPPV)	129 (59%)	90(41%)	59 (27%)	160 (73%)
Cerebrovascular accident	172 (79%)	47 (21%)	126 (58%)	93 (42%)
Cervicogenic dizziness	126 (58%)	93 (42%)	81 (37%)	138 (63%)
Functional dizziness	102 (47%)	117 (53%)	73 (33%)	146 (67%)
Mal de Debarquement	18 (8%)	201 (92%)	14 (6%)	205 (94%)
Meniere’s disease	74 (34%)	145 (66%)	27 (12%)	192 (88%)
Multiple sclerosis	194 (89%)	25 (11%)	157 (72%)	62 (28%)
Perilymphatic fistula	28 (13%)	191 (87%)	17 (8%)	202 (92%)
Persistent postural–perceptual dizziness (PPPD)	54 (25%)	165 (75%)	31 (14%)	188 (86%)
Post-concussion	121 (55%)	98 (45%)	67 (31%)	152 (69%)
Presbystasis	18 (8%)	201 (92%)	13 (6%)	206 (94%)
Traumatic brain injury	193 (88%)	26 (12%)	153 (70%)	66 (30%)
Unilateral vestibular hypofunction	69 (32%)	150 (68%)	44 (20%)	175 (80%)
Vestibular migraine	99 (45%)	120 (55%)	53 (24%)	166 (76%)
Vestibular neuritis	71 (42%)	148 (68%)	40 (18%)	179 (82%)
Vestibular paroxysmia	58 (26%)	161 (74%)	31 (14%)	188 (86%)

* Responses are presented as *n* (%). † Know: The clinician knows about the vestibular disorder. Do not know: The clinician does not know about the mentioned vestibular disorder. Treat: The clinician will treat the vestibular disorder. Do not treat: The clinician will not treat the vestibular disorder.

**Table 8:** The association between different variables (*n* = 219).

	Responders with VRT Experience No. (%)	Responders with No VRT Experience No. (%)	*p*-Value *
Total	59 (27%)	160 (73%)	
Highest earned Degree:			0.66
PT Bachelor’s	39 (66%)	96 (60%)	
Doctor of Physical Therapy (DPT)	4 (7%)	14 (9%)	
Master’s of Science Degree	15 (25%)	42 (26%)	
Terminal Doctorate (PhD, DSc, EdD)	1 (2%)	8 (5%)	
Primary work setting:			0.083
Military/National Guard/Security Forces hospital	6 (10%)	26 (16%)	
Ministry of Health hospital	28 (48%)	72 (45%)	
Private hospital/clinic	19 (32%)	36 (23%)	
Private practice	2 (3%)	1 (1%)	
University hospital	1 (2%)	18 (11%)	
Others	3 (5%)	7 (4%)	
Years of experience in PT field:			0.061
<1 year	4 (7%)	21 (13%)	
1–5 years	30 (51%)	53 (33%)	
6–10 years	14 (24%)	37 (23%)	
>10 years	11 (18%)	49 (31%)	
Have physicians ever referred patients with vestibular disorders to you?			<0.001
Yes	32 (54%)	21 (13%)	
No	27 (46%)	139 (87%)	

* *p*-value of chi-square test.

## Data Availability

Data related to this study are available from the author upon request.

## Abbreviations

The following abbreviations are used in this manuscript:

VRT	Vestibular rehabilitation therapy
PT	Physical therapy
PhD	Doctor of philosophy
DSc	Doctor of science
EdD	Doctor of education
DPT	Doctor of physical therapy
PPPD	Persistent postural–perceptual dizziness
BPPV	Benign paroxysmal positional vertigo
VOR	Vestibulo-ocular reflex
mCTSIB	Modified clinical test of sensory interaction and balance

## Appendix A

We would like first to know about your experience, so please answer the following questions:

**Clinical experience:**

1- How many years’ of experience do you have in your field?

□ Less than a year

□ 1–5 years

□ 6–10 years

□ Over 10 years

2- Do you have any experience in vestibular rehabilitation?

	□ Yes (go to Q 2.1)
	□ No (go to Q 2.2)
	2.1. How many years of experience do you have working with persons with vestibular disorders?

□ Less than a year

□ 1–5 years

□ 6–10 years

□ Over 10 years

2.2. Why don’t you treat patients with vestibular disorders? (check all that apply):

□ Lack of knowledge

□ Lack of confidence

□ Lack of experience

□ Physicians do not refer patients to me

□ Persons with dizziness are managed by another specialty e.g. audiologist, Ear, Nose and Throat physicians

□ I am not allowed to treat persons with dizziness in my practice

□ Other reasons (please describe): …………………………………

3- Have physicians ever referred patients with vestibular disorders to you?

□ Yes

□ No

4- Do you see one patient with vestibular disorder or more per month?

	□ Yes (Go to 4.1)
	□ No
	4.1. What is the average of patients with vestibular disorders do you see per month?

□ 1
□ 2–5
□ 6–10
□ 0ver 10

**Now we will ask you some questions about your beliefs and knowledge**

**Beliefs**

1- Do you believe that physical therapists should be a member of the multidisciplinary team that manages patients with vestibular disorders?

□ Yes

□ Not sure

□ No

2- Do you believe that physical therapists should be more involved in the treatment of patients with vestibular disorders?

□ Yes

□ Not sure

□ No

3- Do you believe that vestibular disorders have a significant impact on the health of individual and society?

□ Yes

□ Not sure

□ No

4- Do you believe that physical therapists (with proper vestibular rehabilitation training) can evaluate patients with vestibular disorders?

□ Yes
□ Not sure
□ No

**Knowledge**

1- Are you confident in communicating with other medical team members (e.g. Ear, Nose and Throat physicians) in managing patients with dizziness?

□ Yes

□ Not sure

□ No

2- Have you learned didactic or practical information/experience about how to treat people with vestibular disorders?

□ Yes (Go to 2.1)

□ Not sure

□ No

2.1. Where did you get your knowledge about vestibular rehabilitation? (click on all that apply to you)

□ In my physical therapy curriculum as part of my entry level education in Saudi Arabia.

□ In my physical therapy curriculum as part of my entry level education outside Saudi Arabia

□ During my clinical experience as a staff member.

□ During my internship as a student in Saudi Arabia

□ During my internship as a student outside Saudi Arabia

□ Through workshops and conferences

□ Got the experience from an expert colleague

□ Other ways (please briefly explain): ……………………………….

3- Do you use frequently vestibular assessment techniques in your clinical practice?

	□ Yes
	□ No
	3.1. Which of the following assessment techniques are you confident in implementing in your clinical practice? (click on all that apply to you)

□ Dix-Hallpike test

□ Supine roll test

□ Side-lying test

□ Head Impulse Test (Head Thrust Test)

□ Dynamic Visual Acuity Test

□ Oculomotor examination (pursuits, saccades, gaze holding)

□ Head Shaking Nystagmus test

□ Perilymphatic fistula test

□ VOR Cancellation

□ Modified Clinical Test of Sensory Interaction and Balance (M-CTSIB)

4- If you have a patient with vestibular disorder, do you use vestibular treatments/interventions in your clinical practice?

	□ Yes
	□ No
	4.1. Which of the following treatment techniques are you confident in implementing in your clinical practice? (click on all that apply to you)

□ Canalith Repositioning Maneuver

□ Semont Maneuver

□ Gufoni Maneuver

□ Barbecue Roll Maneuver

□ Imaginary target exercise

□ Vestibulo-ocular reflex 1 (VOR 1)

□ Vestibulo-ocular reflex 2 (VOR 2)

□ Gaze shifts between two targets

□ Optokinetic stimulation

5- Please determine whether you know or you do not know about the following conditions:
  **Condition**	**Know**	**Do Not Know**
  Benign paroxysmal positional vertigo (BPPV)		
  Cerebrovascular accident		
  Cervicogenic dizziness		
  Functional dizziness		
  Mal de Debarquement		
  Meniere’s disease		
  Multiple sclerosis		
  Perilymphatic fistula		
  persistent postural-perceptual dizziness (PPPD)		
  Post-concussion		
  Presbystasis		
  Traumatic brain injury		
  Unilateral vestibular hypofunction		
  Vestibular migraine		
  Vestibular neuritis		
  Vestibular paroxysmia		

6- Please determine whether you treat or you do not treat the following conditions:
  **Condition**	**Treat**	**Do Not Treat**
  Benign paroxysmal positional vertigo (BPPV)		
  Cerebrovascular accident		
  Cervicogenic dizziness		
  Functional dizziness		
  Mal de Debarquement		
  Meniere’s disease		
  Multiple sclerosis		
  Perilymphatic fistula		
  persistent postural-perceptual dizziness (PPPD)		
  Post-concussion		
  Presbystasis		
  Traumatic brain injury		
  Unilateral vestibular hypofunction		
  Vestibular migraine		
  Vestibular neuritis		
  Vestibular paroxysmia		

7- What percent of your time each month is spent treating various diagnoses (total percentage has to equal 100%)?

□ Musculoskeletal Conditions e.g. low back pain, neck pain: …………%
□ Neurological conditions e.g. stroke, multiple sclerosis, Parkinson disease, spinal cord injury etc. …………%
□ Pediatric conditions e.g. cerebral palsy, Erb’s palsy etc: …………%
□ Vestibular conditions e.g. Benign Paroxysmal Positional Vertigo, vestibular hypofunction, vestibular migraine
□ Sports injuries: …………%
□ Oncology: …………%
□ Cardiopulmonary conditions: …………%
□ Women health: …………%

**Thanks for your patience. Please answer the final part, which is related to your personal information:**

**Personal information:**

Nationality:
□ Saudi
□ Syrian
□ Jordanian
□ Indian
□Filipino
□ Egyptian
□ Other: ………………….
Sex:
□ Male
□ Female

**Educational background:**

Degree:
□ Bachelor
□ Masters
□ Doctor of Physical Therapy (DPT)
□ Doctor of Philosophy (PhD), Doctor of Science (PhD), or Doctor of Education (EdD)

**Work background**

What kind of hospital do you work in?

□ Ministry of Health (MOH) hospital

□ University hospital

□ Private hospital/clinics

□ Private practice

□ Military/National Guard/ Security Forces hospital

□ Other:…………….
